# Screening and Brief Intervention in Prenatal Care Settings

**Published:** 2004

**Authors:** Grace Chang

**Affiliations:** Grace Chang, M.D., M.P.H., is an associate professor of psychiatry at Harvard Medical School and an associate physician in psychiatry and director of education at the Center for Clinical Investigation, Brigham and Women’s Hospital, both positions in Boston, Massachusetts

**Keywords:** pregnancy, prenatal alcohol exposure, identification and screening for AOD (alcohol and other drug) use, CAGE Questionnaire, T-ACE, brief intervention

## Abstract

Pregnant women continue to drink despite evidence that prenatal alcohol consumption can negatively affect fetal growth and development. Because no universally safe level of prenatal alcohol use has been established, it is beneficial to identify and modify a woman’s prenatal alcohol use early in her pregnancy, particularly as her past drinking habits can predict her drinking levels during pregnancy. Some women may voluntarily disclose the extent of their prenatal alcohol consumption. If not, the T-ACE, a four-item screening questionnaire based on the CAGE assessment tool, has been demonstrated to be a valuable and efficient method for identifying a range of alcohol use. Studies have shown that combined with brief interventions, early identification of a woman’s prenatal alcohol use could avert its more severe adverse consequences and may be the logical first-line approach.

According to the U.S. Surgeon General’s most recent advisory, no level of alcohol consumption by pregnant women can be considered safe. A woman drinking alcohol at any time during her pregnancy, even during the earliest weeks, increases the risk of her fetus developing alcohol-related birth defects (see the textbox) (Office of the Surgeon General 2005). Despite the accumulating evidence, pregnant women continue to drink. This article examines the prevalence of alcohol use among pregnant women and the importance and difficulties of identifying a pregnant woman’s level of alcohol consumption. It also describes the implementation of the T-ACE questionnaire as an effective screening instrument for prenatal alcohol consumption, outlines the usefulness of brief interventions with pregnant women, and reviews studies that investigate the use of brief interventions with pregnant women who screen positive on the T-ACE.

## Prevalence of Alcohol Consumption Among Pregnant Women

Women who drink during pregnancy come from all walks of life—and in fact, those who are older (at least 35), non-Hispanic, well educated (beyond high school), and who have a higher household income are more likely to drink while pregnant (CDC 2002). The prevalence of any alcohol use by pregnant women was 12.8 percent in 1999, with 2.7 percent reporting frequent drinking (defined as more than seven drinks per week) and 3.3 percent reporting binge drinking (defined as five or more drinks per episode) (CDC 2002).

The Pregnancy Risk Assessment Monitoring System covering 2000 and 2001 found the overall prevalence of alcohol use in pregnancy to range from 3.4 percent to 9.9 percent in eight States (Phares et al. 2004). Although these figures represent an improvement over the 1988 baseline rate of 21 percent of pregnant women using alcohol, they still fall short of satisfying both the Healthy People 2000 and Healthy People 2010 goals of 94-percent abstinence from alcohol during pregnancy.^1^

## Identifying a Pregnant Woman’s Level of Alcohol Consumption

In accord with the U.S. Surgeon General’s advisory, both the American Academy of Pediatrics and the American College of Obstetricians and Gynecologists recommend that pregnant and preconceptional women be abstinent (Sokol et al. 2003). Thus, it is important to be able to identify and modify a woman’s prenatal alcohol use early in her pregnancy, particularly as her past drinking habits can predict her drinking levels during pregnancy (Russell et al. 1994).

**Surgeon General’s Advisory on Alcohol Use in Pregnancy**

Thirty-two years ago, United States researchers first recognized fetal alcohol syndrome (FAS). FAS is characterized by growth deficiencies (or decreased growth), abnormal facial features (specific facial features), and central nervous system (or brain) abnormalities. FAS falls under the spectrum of adverse outcomes caused by prenatal alcohol exposure called Fetal Alcohol Spectrum Disorders (FASD). The discovery of FAS led to considerable public education and awareness initiatives informing women to limit the amount of alcohol they consume while pregnant. But since that time, more has been learned about the effects of alcohol on a fetus. It is now clear that no amount of alcohol can be considered safe.

I now wish to emphasize to prospective parents, healthcare practitioners, and all childbearing-aged women, especially those who are pregnant, the importance of not drinking alcohol if a woman is pregnant or considering becoming pregnant.

Based on the current, best science available we now know the following:

- Alcohol consumed during pregnancy increases the risk of alcohol related birth defects, including growth deficiencies, facial abnormalities, central nervous system impairment, behavioral disorders, and intellectual development.
- No amount of alcohol consumption can be considered safe during pregnancy.
- Alcohol can damage a fetus at any stage of pregnancy. Damage can occur in the earliest weeks of pregnancy, even before a woman knows that she is pregnant.
- The cognitive deficits and behavioral problems resulting from prenatal alcohol exposure are lifelong.
- Alcohol-related birth defects are completely preventable.

**For these reasons:**

1. A pregnant woman should not drink alcohol during pregnancy.
2. A pregnant woman who has already consumed alcohol during her pregnancy should stop in order to minimize further risk.
3. A woman who is considering becoming pregnant should abstain from alcohol.
4. Recognizing that nearly half of all births in the United States are unplanned, women of child-bearing age should consult their physician and take steps to reduce the possibility of prenatal alcohol exposure.
5. Health professionals should inquire routinely about alcohol consumption by women of child-bearing age, inform them of the risks of alcohol consumption during pregnancy, and advise them not to drink alcoholic beverages during pregnancy.

***Background***

In the United States, FAS is the leading preventable birth defect with associated mental and behavioral impairment. There are many individuals exposed to prenatal alcohol who, while not exhibiting all of the characteristic features of FAS, do manifest lifelong neurocognitive and behavioral problems arising from this early alcohol exposure. In the United States, the prevalence of FAS is between 0.5 and 2 cases per 1,000 births. It is estimated that for every child born with FAS, three additional children are born who may not have the physical characteristics of FAS but still experience neurobehavioral deficits resulting from prenatal alcohol exposure that affect learning and behavior.

The outcomes attributable to prenatal alcohol exposure for the children of women whose alcohol consumption averages seven to 14 drinks per week include deficits in growth; behavior; neurocognition (such as problems in arithmetic, language, and memory); visual-spatial abilities; attention; and deficits in speed of information processing. Patterns of exposure known to place a fetus at greatest risk include binge drinking, defined as having five or more drinks at one time, and drinking seven or more drinks per week.

Despite public health advisories and subsequent efforts to disseminate this information, including a Surgeon General’s advisory in 1981, recent data indicate that significant numbers of women continue to drink during pregnancy, many in a high-risk manner that places the fetus at risk for a broad range of problems arising from prenatal alcohol exposure, including fetal alcohol syndrome. For example, data suggest that rates of binge drinking and drinking seven or more drinks per week among both pregnant women and non-pregnant women of childbearing age have not declined in recent years. Many women who know they are pregnant report drinking at these levels.

In addition, recent analysis of obstetrical textbooks suggests that physicians may not be receiving adequate instruction in the dangers of prenatal alcohol exposure. The American College of Obstetricians and Gynecologists advises against drinking at all during pregnancy. Nevertheless, only 24 percent of obstetrical textbooks published since 1990 recommended abstinence during pregnancy, despite 30 years of research since the first publications proposed a link between alcohol exposure and birth defects. Scientific evidence amassed in these decades has fortified the rationale for the original advisory against alcohol consumption during pregnancy. Continuing research has generated a wealth of new knowledge on the nature of fetal alcohol-induced injury, the underlying mechanisms of damage, concurrent risk factors, and the clinical distinction of alcohol-related deficits from other disorders.

Alcohol-related birth defects are completely preventable. A number of resources are available to assist healthcare and social services professionals in advising their patients to reduce and refrain from alcohol in pregnancy. These resources include the National Institute on Alcohol Abuse and Alcoholism, NIH (http://www.niaaa.nih.gov), the Centers for Disease Control and Prevention (www.cdc.gov/ncbddd/fas), and the Substance Abuse and Mental Health Services Administration (www.fascenter.samhsa.gov/).

SOURCE: Office of the Surgeon General. Press Release: “U.S. Surgeon General Releases Advisory on Alcohol Use in Pregnancy, February 21, 2005.” Available at: www.hhs.gov/surgeongeneral/pressreleases/sg02222005.html.

Ascertaining a woman’s prenatal alcohol consumption poses several challenges. First, many women will reduce their alcohol consumption once they learn they are pregnant (Smith et al. 1987), but a woman may have been drinking harmful amounts of alcohol prior to detecting her pregnancy. Therefore, asking a pregnant woman standard questions about her current quantity and frequency of alcohol use may not provide accurate information. Asking a woman about her drinking patterns before she became pregnant could elicit more accurate measures of her first-trimester drinking (Day et al. 1993).

Second, women may under-report their consumption because they are embarrassed or afraid to admit that they are drinking during pregnancy (Jacobson et al. 2002), or they may believe that small amounts of alcohol are inconsequential.

Third, popular screening instruments such as the CAGE or SMAST have been tested in other populations (e.g., heavy-drinking males), so they may be less accurate in identifying risk drinking by pregnant women (Bradley et al. 1998).

The T-ACE, a four-item questionnaire based on the CAGE, is a simple screening instrument. It can identify lifetime alcohol use; it also can identify prenatal consumption and has been tested in diverse obstetric samples (Sokol et al. 1989; Chang et al. 1998). Moreover, it has proven to be a valuable and efficient tool for identifying a range of alcohol use, including any current prenatal alcohol consumption, prepregnancy risk drinking defined as more than two drinks per drinking day, and lifetime alcohol diagnoses based on the *Diagnostic and Statistical Manual of Mental Disorders, Third Edition, Revised* (Chang et al. 1998).

Clinicians may wish to consider other screening instruments, such as the TWEAK, which has been studied among pregnant women (Flynn et al. 2003). However, the level of at-risk drinking identified in the TWEAK is double the currently accepted definition of one drink per day (Sokol et al. 2003). Moreover, the TWEAK, when compared with the AUDIT and AUDIT-C (neither of which has been well studied with prenatal populations), had low sensitivity as an alcohol screening questionnaire among female Veterans Affairs outpatients (Bush et al. 2003).

**At a Glance**

**Alcohol Consumption Among Pregnant Women**

- 12.9 percent of pregnant women reported some level of alcohol use in 1999.
- 2.7 percent of pregnant women reported frequent drinking in 1999.
- 3.3 percent of pregnant women reported binge drinking in 1999.
- 3.4–9.9 percent of pregnant women in eight States reported using alcohol during pregnancy in 2000 and 2001.

SOURCE: Centers for Disease Control and Prevention (CDC) 2002.

### The T-ACE Questionnaire

The T-ACE questions are:

**Table:** 

**T**	**Tolerance:** How many drinks does it take to make you feel high?
**A**	Have people **annoyed** you by criticizing your drinking?
**C**	Have you ever felt you ought to **cut down** on your drinking?
**E**	**Eye-opener:** Have you ever had a drink first thing in the morning to steady your nerves or get rid of a hangover?

The T-ACE is considered to be positive with a score of 2 or more. Affirmative answers to the A, C, and E questions are each scored 1 point. A reply of more than two drinks to the T question is scored 2 points (Sokol et al. 1989).

## Brief Interventions

If a pregnant woman discloses that she currently is drinking alcohol, drank more than the NIAAA sensible drinking limits prior to pregnancy (more than seven standard drinks a week or more than three drinks at a time), or scores positive on the T-ACE, the clinician should parlay the results into an opportunity to discuss her prenatal alcohol use and tell her that there is no safe drinking limit during pregnancy. If appropriate, the clinician may refer her for more in-depth assessment, or offer her a brief intervention (BI) to modify her prenatal alcohol use (Chang 2001).

Brief interventions have been recommended as the first step for approaching people with mild-to-moderate alcohol problems. Pregnant women generally are motivated to change their behaviors and only infrequently have severe alcohol problems (Hankin et al. 2000), so they may be especially receptive to receiving BIs. Thus, early identification of a woman’s potentially problematic alcohol consumption and intervention could avert the more severe adverse consequences of prenatal alcohol use.

BIs typically include four steps: providing assessment and direct feedback, establishing contracts and setting goals, using behavioral modification techniques, and distributing pamphlets or other handouts for self-help and reinforcement. Brief interventions have proven effective (see the article by Moyer and Finney in the companion issue). Their advantages include the following:

- They generally are well accepted by people with less severe alcohol problems.
- They can be given by a variety of providers in a broad range of clinical settings.
- They are cost-effective.

In general, people change their drinking behaviors in the 6 months following the intervention (Wutzke et al. 2002; Moyer et al. 2002). Most pregnant women seek prenatal care during their first trimester. Ideally, women will stop drinking prior to becoming pregnant, but offering an intervention during the first trimester is the next-best option.

## Studies of the Effectiveness of BIs in Prenatal Settings

Research studies have systematically examined the effect of brief interventions for pregnant women in prenatal settings. For example, two randomized trials have been conducted with pregnant women who screened positive on the T-ACE while receiving care at the obstetric practices of the Brigham and Women’s Hospital in Boston. In the first study, 250 pregnant women who screened positive on the T-ACE and reported consuming alcohol in the previous 6 months were asked to complete a comprehensive assessment of their drinking histories. Half of these women then were randomized to receive a brief intervention. With 99-percent postpartum followup, investigators found that women in both groups reduced their prenatal alcohol use after enrolling in the study. However, among those who were abstinent at study enrollment, participants randomly assigned to the brief intervention group were more likely to maintain their abstinence (*p* < .05) (Chang et al. 1999; Chang et al. 2000).

In the second study, 304 pregnant women, selected because they screened positive on the T-ACE and were considered at risk for prenatal alcohol use, were randomized with a support partner of their choice (usually husbands or biological fathers of their unborn children) to receive a brief intervention or not. Results indicated that the women with the highest levels of drinking at enrollment had the greatest reductions in drinking when they received the brief intervention (*p* = .01). Moreover, the effects of the brief intervention were significantly enhanced when a partner participated (*p* = .05) (Chang et al. 2005).

Another innovative approach in the prenatal setting, the Protecting the Next Pregnancy Project, focused on women who were identified as drinking during their last pregnancy (called the index pregnancy). The goal of the study was to reduce prenatal alcohol use in the women’s *next* pregnancies (Hankin et al. 2000; Hankin and Sokol 1995).

Three hundred women who consumed at least four drinks per week at the time of conception for the index pregnancy were randomized 4 weeks after giving birth to receive an intensive brief intervention or to a control group that received standard care. They were followed for up to 5 years. Approximately one-third gave birth to one or more children in the followup period. Women who received the brief intervention drank significantly less than those in the control group during their subsequent pregnancies. The women who drank less also had better birth outcomes, with fewer low-birth-weight babies and fewer premature deliveries. Moreover, children born to mothers in the brief intervention group showed better neurobehavioral performance at 13 months than the control group children (Hankin 2002). (For more information about the effects of maternal alcohol consumption on child behavior, see Sood et al. 2001, O’Connor and Whaley 2003, and Whaley and O’Connor 2003.)

## Conclusion

Screening a woman for alcohol use with a validated questionnaire during her pregnancy can provide important information about what steps should be taken to assist her in modifying her drinking behavior, which might otherwise not be disclosed. If appropriate, clinicians then can provide assessment and brief intervention to help eliminate, or at least reduce, her prenatal alcohol use, minimize fetal risk, and maximize pregnancy outcome.

## Footnotes

1 The data reflect patterns of consumption for eight States only and are not generalizable across the country.
